# Picturing the Multiple Sclerosis Patient Journey: A Symptomatic Overview

**DOI:** 10.3390/jcm13195687

**Published:** 2024-09-25

**Authors:** Inês Correia, Catarina Bernardes, Carolina Cunha, Carla Nunes, Carmo Macário, Lívia Sousa, Sónia Batista

**Affiliations:** 1 Neurology Department, Hospitais da Universidade de Coimbra, Unidade Local de Saúde de Coimbra, 3004-561 Coimbra, Portugalúde.pt (C.N.);; 2 Faculty of Medicine, University of Coimbra, 3000-548 Coimbra, Portugal

**Keywords:** multiple sclerosis, presentations, symptoms, diagnosis

## Abstract

**Background**: Multiple sclerosis (MS) presents a wide range of clinical symptoms, historically understood through long-term studies of earlier patient cohorts. However, due to improved diagnostic criteria, modern patients are diagnosed earlier and benefit from effective treatments, altering the disease’s natural history. This study aimed to assess the clinical symptoms of MS patients in a modern population at various stages: before diagnosis, at diagnosis, during the disease course, and at the time of the survey. **Methods**: This was an observational study with retrospective and cross-sectional components; patients that fulfilled the 2017 revised McDonald criteria for MS completed a survey evaluating demographic and clinical data. **Results**: We included 163 patients, 69.9% female, with a mean age of 48.21 years; 87.1% had relapsing–remitting MS (RRMS), with a median EDSS of 2.0. Before diagnosis, 74.2% of patients experienced symptoms, mainly sensory issues (39.3%), fatigue (29.4%), and imbalance (27%). Motor and coordination symptoms were more common in progressive forms. At diagnosis, sensory (46.6%) and motor complaints (36.8%) were most prevalent. In RRMS and secondary progressive MS (SPMS), sensory and motor complaints predominated alongside imbalance, while primary progressive MS (PPMS) was characterized by motor, imbalance, and genitourinary symptoms. Throughout the disease, sensory symptoms were most common (76.1%), with fatigue (73%) and motor issues (62.6%) more prevalent in progressive forms. At the time of the survey, 50.7% of RRMS patients were asymptomatic, while progressive patients continued to experience motor symptoms, imbalance, and fatigue. **Conclusions**: The study reflects the modern spectrum of MS symptoms, consistent with previous research.

## 1. Introduction

Multiple sclerosis (MS) is the most common chronic inflammatory, demyelinating, and neurodegenerative disease of the central nervous system in young adults, currently being the primary cause of non-traumatic disability in this population [1].

The clinical manifestation of MS varies widely, not only due to the type of disease onset (relapsing or progressive) but also because the symptoms arise as a result of both the location and the size of these lesions, where even a small lesion in a critical area is prone to induce symptoms [1,2,3,4]. Investigating the wide range of symptoms affecting individuals with MS is essential for gaining a comprehensive understanding of the challenges they face throughout the disease’s course. MS is renowned for its diverse impact on various aspects of daily life, spanning physical abilities, cognitive functions, and emotional well-being.

While there are no pathognomonic clinical findings in MS, certain manifestations are notably distinct to this condition [1]. In most cases, nearly 85% of the patients, the onset of MS is marked by an initial clinical episode, defined as clinically isolated syndrome (CIS), in which criteria for definitive MS are unmet. CIS can be mono- or polysymptomatic and is contingent upon the specific sites of demyelinating lesions within the CNS. It can involve the optic nerve (optic neuritis), spinal cord (myelitis), brainstem or cerebellum (brainstem and/or cerebellar syndromes), or the cerebral hemispheres (cerebral hemispheric syndrome) [1,5,6,7,8,9].

Optic neuritis is the first manifestation of the disease in about 25% of the patients and occurs at some point in the course of the disease in about 70% of patients with MS [1,9,10,11,12]. Sensory symptoms are the first disease manifestation in up to 43% of patients and are present in almost every patient at some time during the course of disease [1,9,13,14]. Motor symptoms are the first signs in 30–40% of patients and are experienced by nearly all individuals as the disease progresses, playing a significant role in causing physical disability [1,9,14]. Brainstem and cerebellar manifestations are very common in MS, being present in up to 70% of patients during their lifetime, and are the first manifestation in nearly 20% of the patients [1,9,15,16,17,18].

The primary source of our knowledge regarding the clinical presentation of MS originates from early studies involving the collection of large numbers of cases in historical cohorts, allowing for the description of the overall course and prognosis of MS. These studies involved tracking patients over extended periods, the majority of whom remained untreated for years since there were no treatments available, allowing us to understand the disease’s evolution and ascertaining prognoses. Nonetheless, it is important to note that these patients received their diagnoses based on earlier diagnostic criteria, implying that their diagnoses likely occurred at a later stage in the course of the disease, and as a result, the clinical manifestations observed at presentation and persisting over a lifetime may not fully represent the modern patient population, which benefits from earlier diagnosis and effective treatment. Our objective was to evaluate the clinical symptoms experienced by the patients in a modern-day population before the diagnosis was made, at MS diagnosis, throughout the disease, and at the moment of the survey, acknowledging both the shared and distinct features among different disease subtypes. Comprehending these symptoms is critical for healthcare professionals, researchers, and patients themselves to enhance the management and overall quality of life for those living with MS.

## 2. Materials and Methods

This is an observational study, incorporating both retrospective and cross-sectional components. The retrospective elements include data on symptoms prior to MS diagnosis, at diagnosis, and throughout the disease up to the time of the survey, as well as the age at symptoms onset and diagnosis. The cross-sectional components encompass current age, present symptoms, and disability status.

Patients that fulfilled the 2017 revised McDonald criteria [19] for MS and were followed in the Neurology Department of a tertiary hospital were selected for inclusion in the study. The selected patients were personally invited to participate in the study during their visits to Demyelinating Diseases consultations or Neurology Day Hospital sessions between 1 December 2021 and 31 October 2022. This research was conducted in accordance with the World Medical Association’s Declaration of Helsinki. The study protocol was reviewed and approved by the Ethical Committee of the Faculty of Medicine of the University of Coimbra (CE-010/2018) and the Ethics Committee of the Regional Health Administration of Centro Portugal (94/2018). The patients or their respective caregivers received an information form and, upon agreeing to participate, signed and completed an informed consent for the collection and use of data. All collected data were anonymized.

Participants were required to complete a form covering demographics and clinical data related to MS. Data collected comprised demographic data including sex, age at symptoms’ onset and at diagnosis, current age, and clinical data regarding symptoms. Patients were questioned about the presence of symptoms before diagnosis, at the time of diagnosis, during the course of the disease, and at the present moment. The symptoms assessed covered the following areas: sensory symptoms (paresthesia), motor symptoms (weakness), imbalance or discoordination, blurry vision or ocular pain, diplopia (double vision), headache, trigeminal neuralgia or other facial pain, facial asymmetry or facial spasm, vertigo or dizziness, dysarthria, dysphagia, hearing impairment, nystagmus-related complaints/oscillopsia, cognitive complaints, depression (depressive symptoms), fatigue, genitourinary complaints, and gastrointestinal symptoms. The disability score assessed by the Expanded Disability Status Scale (EDSS) and MS subtype was then introduced by the investigator based on information in the most recent consultation from the patient’s hospital data file.

Regarding demographic characteristics, continuous variables were expressed as mean plus standard deviation, ordinal variables were expressed as median plus interquartile range, and frequencies and percentages were used for categorical variables. The Kruskal–Wallis H test was used to compare MS subtypes. Statistical differences were considered significant if *p* < 0.05 using a two-sided comparison.

## 3. Results

### 3.1. Demographic and Clinical Characteristics of the Patients

We included 163 patients, 69.9% female, with a mean age of 48.21 years. Most patients had RRMS (87.1%), with a median EDSS of 2.0.

The demographic and clinical characteristics of the total population are presented in Table 1. RRMS patients in our cohort were significantly younger compared to progressive forms, not only at the time of the survey (48.21 vs. 59.75 to 64.47 years) but also when they were diagnosed (35.54 vs. 43 to 44.65 years); they had a shorter disease duration (8 vs. 14 to 19 years) and were considerably less disabled (1.5 vs. 6.25 to 6.5).

### 3.2. Symptoms Experienced before MS Diagnosis

**Figure 1:**
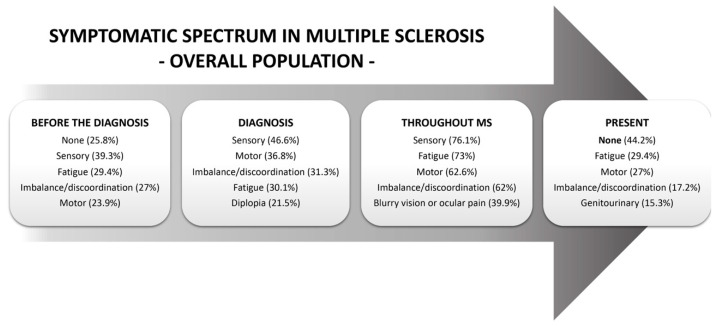
Symptomatic spectrum in MS in the entire population. The five most prevalent symptoms in the overall population across the various time points are illustrated. Before diagnosis, most patients had already experienced some symptoms, mainly sensory complaints and fatigue. At the time of diagnosis, sensory and motor symptoms were the most common presentations. As the disease progressed, the range of symptoms expanded, with many patients also experiencing issues such as imbalance, incoordination, and visual disturbances. However, these symptoms were not permanent for the majority of patients, and at the time of the survey, nearly half of the population was asymptomatic. Abbreviations: MS—multiple sclerosis.

**Figure 2:**
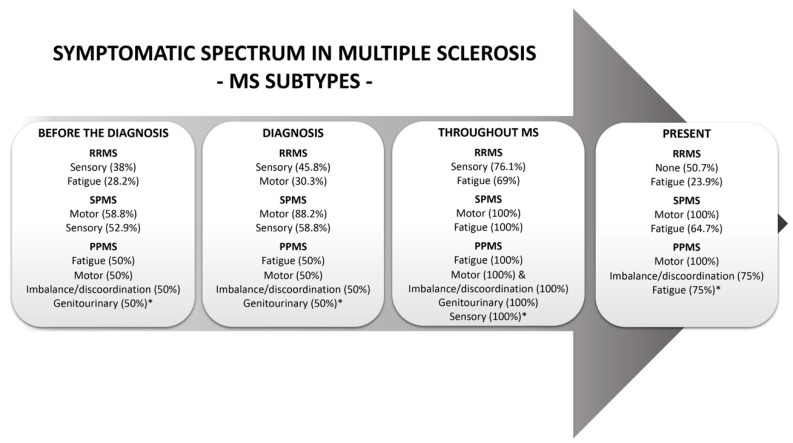
Symptomatic spectrum in MS subtypes. The two most prevalent symptoms based on MS subtypes are illustrated. In RRMS, sensory complaints are the most frequent symptoms, occurring before diagnosis in many cases, in nearly half of patients at the time of diagnosis, and in most patients throughout the disease. In contrast, motor impairment and fatigue are the most common symptoms in SPMS and PPMS patients. At the time of the survey, half of the patients with RRMS were asymptomatic, whereas all SPMS and PPMS patients experienced motor impairment, with the majority also reporting fatigue. Abbreviations: MS—multiple sclerosis; RRMS—relapsing–remitting multiple sclerosis; SPMS—secondary progressive multiple sclerosis; PPMS—primary progressive multiple sclerosis. * Due to identical percentages, the number of symptoms selected was adjusted.

Prior to being diagnosed with MS, only 25.8% of patients did not experience any symptoms (Table 2). The most frequent symptoms across all subtypes of MS present before the diagnosis were sensory symptoms (39.3%), fatigue (29.4%), imbalance or discoordination (27%), and motor symptoms (23.9%) (Table 2). The latter two categories of symptoms were particularly frequent in SPMS and PPMS patients (Table 2). Genitourinary symptoms were already experienced by half of the patients with PPMS. The complete list of symptoms experienced before the diagnosis of MS in the overall population, as well as according to MS phenotype, is available in Table 2. The most prevalent symptoms across various time points are highlighted in the figures. Figure 1 highlights the most prevalent symptoms in the overall population across the various time points, while Figure 2 highlights the most common symptoms based on MS subtypes.

### 3.3. Symptoms at Clinical Presentation Leading to MS Diagnosis

The most frequent symptoms experienced by the total population of MS patients at diagnosis were sensory (46.6%) and motor complaints (36.8%), imbalance or discoordination (31.3%) (Table 3). When different subtypes of MS were considered, the frequencies of these symptoms varied, except for fatigue (30.1%), which was importantly present in all subtypes of patients, especially in the progressive subtypes (Table 3). Of note, depression was already experienced by 23.5% of the SPMS patients and 25% of PPMS patients.

In the RRMS group, most patients experienced sensory (45.8%) and motor symptoms (30.3%), imbalance or discoordination (31.3%), but also diplopia (21.8%) and blurry vision or ocular pain (20.4%) (Table 3).

In the SPMS group, most patients experienced motor symptoms (88.2%), followed by sensory complaints (58.8%), and imbalance or discoordination (52.9%). Diplopia, symptoms of vertigo and dizziness, and genitourinary complaints were also present in 23.5% of the patients, respectively (Table 3).

In PPMS group, most patients (50% each) experienced motor symptoms, imbalance, and discoordination, as well as genitourinary complaints. Trigeminal neuralgia was also more frequent in PPMS (Table 3).

The complete list of symptoms experienced at clinical presentation leading to MS diagnosis in the overall population and according to MS phenotype is available in Table 3.

### 3.4. Symptoms Experienced Throughout the Course of MS

Throughout the course of MS, patients experienced a wide range of diverse symptoms.

Sensory symptoms were uniformly experienced by all subtypes of MS (76.1%, ranging from 70.6% to 100%) (Table 4). 

Fatigue (73%, ranging from 69% to 100%), motor complaints (62.6%, ranging from 57% to 100%), imbalance or discoordination (62.6%, ranging from 57.7% to 100%), genitourinary symptoms (36.2%, ranging from 29.6% to 100%), depression (28.2%, ranging from 24.6% to 52.9%), and trigeminal neuralgia (9.2%, ranging from 7% to 25%), were significantly more frequent in progressive forms. Cognitive complaints were more frequent in SPMS patients (52.9%) (Table 4). The complete list of symptoms experienced throughout the course of MS in the overall population and according to MS phenotype is available in Table 4.

### 3.5. Symptoms Encountered at the Time of the Survey

When asked about their current symptoms, 50.7% of RRMS patients reported having no complaints. In the remaining RRMS patients, the major complaint was fatigue (23.9%) (Table 5).

Conversely, all patients with progressive forms of the disease experienced symptoms, especially motor complaints (100%), imbalance or discoordination (ranging from 41.2% to 75%), and fatigue (ranging from 64.7% to 75%) (Table 5). The complete list of symptoms experienced at the time of the survey in the overall population and according to MS phenotype is available in Table 5.

## 4. Discussion

Understanding the diverse range of symptoms associated with MS is crucial for both early diagnosis and effective disease management. Given the recent significant changes in both diagnostic criteria and treatment approaches, it is important to review the symptom spectrum over the disease’s evolution.

MS diagnostic criteria have evolved over time, aiming for earlier diagnosis. Over the years, paraclinical findings have been incorporated into the diagnostic criteria, replacing the need for a second clinical relapse and reducing the time to diagnosis. However, in our cohort, despite using the 2017 revised McDonald criteria, only 25.8% of our patients had no reported symptoms before the diagnosis of MS was made. The most frequently reported symptoms across all MS subtypes were sensory issues, fatigue, and imbalance/incoordination. Although these are classical symptoms of MS, they are non-specific and may be difficult to identify, often being undervalued by both patients and clinicians. While these symptoms may reflect previously unrecognized relapses, they could also occur during a prodromal phase of the disease, as hypothesized in the literature. [20]. This more recently described stage may also be related to depression, cognitive difficulties, fatigue, and genitourinary or gastrointestinal symptoms, which were reported by many patients before diagnosis [21].

In alignment with the previous literature, sensory symptoms were the most commonly reported both at diagnosis and throughout the course of the disease among all MS subtypes [1,9,13,14,22,23,24,25], followed by motor symptoms, imbalance/ discoordination, and fatigue, particularly in those with progressive subtypes [1,9,14,22,24]. Imbalance/discoordination and fatigue have multiple mechanisms, and fatigue may occur regardless of relapses [1,25,26,27,28]. In our cohort, patients with progressive subtypes were significantly older not only at the time of the survey but also at diagnosis, had a longer disease duration, and were considerably more disabled, which may explain the higher prevalence of these symptoms.

Regarding brainstem and cerebellar manifestations, symptoms can be highly variable. Typical symptoms attributed to brainstem and cerebellar lesions include imbalance/discoordination, vertigo or dizziness, and diplopia [29]. However, other less common brainstem symptoms such as hearing loss, dysarthria, and dysphagia must also be considered [30]. These symptoms were reported similarly in the existing literature [22,23,24,25,30,31,32,33,34,35], with the exception of vertigo. While the existing literature identifies vertigo as an onset symptom in only 5% of patients [22,23,24,25,36], it was reported by 14.7% of our cohort. This may be attributed to the strong multidisciplinary team at our center, which includes neuro-ophthalmology/otology specialists. Vertigo is a very non-specific and sometimes difficult-to-explain symptom that may have a broad etiology and may be undervalued without a detailed and specialized evaluation. Dysphagia is also known to become increasingly significant as disability progresses, affecting almost half of patients, though it is often underreported [24,37]. This could explain the low prevalence of this symptom in our cohort.

Similarly, cognitive complaints were reported by less than one third of patients, although cognitive impairment has been shown to affect a significant proportion of MS patients (40% to 70%, depending on the population studied and the neuropsychological tests used), even in the early stages of the disease [1,4,38,39,40]. This likely reflects patients’ lack of awareness or insight into cognitive deficits that may only be detected through formal neuropsychological assessment.

Affective disturbances, primarily depression, have also been reported in as many as two-thirds of patients [1,41]. However, the social stigma surrounding mental health may have contributed to a reluctance to disclose such sensitive information.

Pain symptoms in MS can have various manifestations and underlying causes [24]. Although less common, trigeminal neuralgia is a highly disabling and challenging-to-treat condition that significantly impacts an individual’s quality of life.

MS is recognized as a chronic, progressive, and highly disabling condition. However, in recent decades, its treatment has evolved dramatically, leading to a paradigm shift in disease management. This likely explains why almost half of our cohort was asymptomatic at the time of the survey. Despite these significant advances in treatment, efforts must continue to further reduce the proportion of symptomatic, impaired patients. This will require not only the development of more effective disease-modifying drugs but also improvements in symptomatic therapy.

Our study has several limitations. First, our cohort predominantly consists of individuals with RRMS (87.1%), which do not accurately represent the entire spectrum of MS patients, leading to a selection bias. Since progressive forms are known to have a different pathophysiology and a more disabling clinical presentation, our findings should not be generalized to these MS subtypes.

Additionally, the limited sample size may not have captured less common symptoms or differences between subtypes, underscoring the need for a larger and more diverse sample to provide a more comprehensive understanding of MS symptoms. Second, data collection was retrospective, relying on patients’ recollections of their symptoms over time, and current treatments, including symptomatic ones, were not evaluated. This approach may have introduced a recall bias, resulting in under- or over-reporting of symptoms, particularly among patients with cognitive impairment. On the other hand, this study relied on self-reported data based on patients’ self-assessments, which may prioritize certain symptoms over others. Finally, this study did not include a control group for comparison, which could help differentiate between symptoms related to MS and those present in the general population.

## 5. Conclusions

In conclusion, our study sheds light on the diverse spectrum of symptoms experienced by individuals with MS diagnosed in the current era. These symptoms encompass a wide range of physical, cognitive, and emotional challenges that significantly impact the quality of life of those affected by the disease. Addressing the multifaceted nature of MS symptoms, tailoring interventions to specific manifestations, and exploring the prodromal phase will contribute to more effective management strategies and ultimately improve the quality of life of those living with this complex neurological condition.

## Figures and Tables

**Table 1:** Demographic and clinical characteristics of the patients.

	Total Population (*n* = 163)	RRMS (*n* = 142)	SPMS (*n* = 17)	PPMS (*n* = 4)	*p*
Female, *n* (%)	114 (69.9%)	97 (68.3%)	14 (82.4%)	3 (75%)	NS
Current age, years (min–max, SD)	48.21 (22–79, ±12.80)	45.94 (22–69, ±11.34)	64.47 (42–79, ±11.88)	59.75 (50–73, ±11.18)	<0.001
Age at symptoms onset, years (min–max, SD)	33.4 (8–64, ±11.87)	32.58 (8–63, ±11.13)	38.47 (12–64, ±14.85)	40.75 (16 -56, ±18.89)	NS
Age at MS diagnosis, years (min–max, SD)	36.67 (12–67, ±11.59)	35.54 (12–63, ±10.78)	44.65 (19–67, ±13.15)	43 (17–60, ±19.88)	0.016
Disease duration, years (min–max, IQR)	10.0 (0–39, ±14)	8 (0–34, ±13)	19 (9–39, ±16)	14 (5–34, ±23)	<0.001
EDSS, median (min–max, IQR)	2 (1–8.5, ±1.5)	1.5 (2–6.5, ±1)	6.5 (3–8.5, ±1.5)	6.25 (5.5–7, ±1.3)	<0.001
MS phenotype RRMS, *n* (%) SPMS, *n* (%) PPMS, *n* (%)	142 (87.1%) 17 (10.4%) 4 (2.5%)	-	-	-	

Abbreviations: RRMS—relapsing–remitting multiple sclerosis; SPMS—secondary progressive multiple sclerosis; PPMS—primary progressive multiple sclerosis; NS—non-significant; min–minimum; max—maximum; SD—standard deviation; MS—multiple sclerosis; IQR—interquartile range.

**Table 2:** Symptoms experienced before the diagnosis of MS in the entire population and according to MS phenotype.

Symptoms	Total Population (*n =* 163)	RRMS (*n* = 142)	SPMS (*n* = 17)	PPMS (*n* = 4)	*p*
None, *n* (%)	42 (25.8)	38 (26.8)	3 (17.6)	1 (25)	NS
Sensory, *n* (%)	64 (39.3)	54 (38)	9 (52.9)	1 (25)	NS
Motor, *n* (%)	39 (23.9)	27 (19)	10 (58.8)	2 (50)	0.001
Imbalance or discoordination, *n* (%)	44 (27)	34 (23.9)	8 (47.1)	2 (50)	NS
Blurry vision or ocular pain, *n* (%)	28 (17.2)	25 (17.6)	2 (11.8)	1 (25)	NS
Diplopia, *n* (%)	19 (11.7)	18 (12.7)	1 (5.9)	0 (0)	NS
Headache, *n* (%)	29 (17.8)	25 (17.6)	3 (17.6)	1 (25)	NS
Trigeminal neuralgia or other facial pain, *n* (%)	3 (1.8)	3 (2.1)	0 (0)	0 (0)	NS
Facial asymmetry or facial spasm, *n* (%)	5 (3.1)	5 (3.5)	0 (0)	0 (0)	NS
Vertigo or dizziness, *n* (%)	24 (14.7)	21 (14.8)	3 (17.6)	0 (0)	NS
Dysarthria, *n* (%)	9 (5.5)	8 (5.6)	1 (5.9)	0 (0)	NS
Dysphagia, *n* (%)	1 (0.6)	1 (0.7)	0 (0)	0 (0)	NS
Hearing impairment, *n* (%)	2 (1.2)	2 (1.4)	0 (0)	0 (0)	NS
Nystagmus-related complaints/oscillopsia, *n* (%)	9 (5.5)	9 (6.3)	0 (0)	0 (0)	NS
Cognitive complaints, *n* (%)	9 (5.5)	7 (4.9)	2 (11.8)	0 (0)	NS
Depression, *n* (%)	14 (8.6)	11 (7.7)	3 (17.6)	0 (0)	NS
Fatigue, *n* (%)	48 (29.4)	40 (28.2)	6 (35.3)	2 (50)	NS
Genitourinary, *n* (%)	11 (6.7)	7 (4.9)	2 (11.8)	2 (50)	0.001
Gastrointestinal, *n* (%)	12 (7.4)	9 (6.3)	2 (11.8)	1 (25)	NS

Abbreviations: MS—multiple sclerosis; RRMS—relapsing–remitting multiple sclerosis; SPMS—secondary progressive multiple sclerosis; PPMS—primary progressive multiple sclerosis; NS—non-significant.

**Table 3:** Symptoms at clinical presentation leading to MS diagnosis in the entire population and according to MS phenotype.

Symptoms	Total Population (*n =* 163)	RRMS (*n* = 142)	SPMS (*n* = 17)	PPMS (*n* = 4)	*p*
Sensory, *n* (%)	76 (46.6)	65 (45.8)	10 (58.8)	1 (25)	NS
Motor, *n* (%)	60 (36.8)	43 (30.3)	15 (88.2)	2 (50)	<0.001
Imbalance or discoordination, *n* (%)	51 (31.3)	40 (28.2)	9 (52.9)	2 (50)	NS
Blurry vision or ocular pain, *n* (%)	33 (20.2)	29 (20.4)	3 (17.6)	1 (25)	NS
Diplopia, *n* (%)	35 (21.5)	31 (21.8)	4 (23.5)	0 (0)	NS
Headache, *n* (%)	24 (14.7)	21 (14.8)	2 (11.8)	1 (25)	NS
Trigeminal neuralgia or other facial pain, *n* (%)	4 (2.5)	2 (1.4)	1 (5.9)	1 (25)	0.007
Facial asymmetry or facial spasm, *n* (%)	8 (4.9)	7 (4.9)	1 (5.9)	0 (0)	NS
Vertigo or dizziness, *n* (%)	24 (14.7)	20 (14.1)	4 (23.5)	0 (0)	NS
Dysarthria, *n* (%)	15 (9.2)	13 (9.2)	2 (11.8)	0 (0)	NS
Dysphagia, *n* (%)	1 (0.6)	1 (0.7)	0 (0)	0 (0)	NS
Hearing impairment, *n* (%)	3 (1.8)	3 (2.1)	0 (0)	0 (0)	NS
Nystagmus-related complaints/oscillopsia, *n* (%)	8 (4.9)	8 (5.6)	0 (0)	0 (0)	NS
Cognitive complaints, *n* (%)	14 (8.6)	11 (7.7)	3 (17.6)	0 (0)	NS
Depression, *n* (%)	18 (11)	13 (9.2)	4 (23.5)	1 (25)	NS
Fatigue, *n* (%)	49 (30.1)	40 (28.2)	7 (41.2)	2 (50)	NS
Genitourinary, *n* (%)	23 (14.1)	17 (12)	4 (23.5)	2 (50)	NS
Gastrointestinal, *n* (%)	11 (6.7)	8 (5.6)	3 (17.6)	0 (0)	NS

Abbreviations: MS—multiple sclerosis; RRMS—relapsing–remitting multiple sclerosis; SPMS—secondary progressive multiple sclerosis; PPMS—primary progressive multiple sclerosis; NS—non-significant.

**Table 4:** Symptoms experienced throughout the course of MS in the entire population and according to MS phenotype.

Symptoms	Total Population (*n =* 163)	RRMS (*n* = 142)	SPMS (*n* = 17)	PPMS (*n* = 4)	*p*
Sensory, *n* (%)	124 (76.1)	108 (76.1)	12 (70.6)	4 (100)	NS
Motor, *n* (%)	102 (62.6)	81 (57)	17 (100)	4 (100)	0.001
Imbalance or discoordination, *n* (%)	101 (62)	82 (57.7)	15 (88.2)	4 (100)	0.015
Blurry vision or ocular pain, *n* (%)	65 (39.9)	60 (42.3)	4 (23.5)	1 (25)	NS
Diplopia, *n* (%)	52 (31.9)	47 (33.1)	5 (29.4)	0 (0)	NS
Headache, *n* (%)	53 (32.5)	46 (32.4)	5 (29.4)	2 (50)	NS
Trigeminal neuralgia or other facial pain, *n* (%)	15 (9.2)	10 (7.0)	4 (23.5)	1 (25)	0.047
Facial asymmetry or facial spasm, *n* (%)	17 (10.4)	16 (11.3)	1 (5.9)	0 (0)	NS
Vertigo or dizziness, *n* (%)	63 (38.7)	51 (35.9)	9 (52.9)	3 (75)	NS
Dysarthria, *n* (%)	33 (20.2)	27 (19)	5 (29.4)	1 (25)	NS
Dysphagia, *n* (%)	7 (4.3)	6 (4.2)	1 (5.9)	0 (0)	NS
Hearing impairment, *n* (%)	12 (7.4)	12 (8.5)	0 (0)	0 (0)	NS
Nystagmus-related complaints/oscillopsia, *n* (%)	28 (17.2)	26 (18.3)	1 (5.9)	1 (25)	NS
Cognitive complaints, *n* (%)	44 (27)	34 (23.9)	9 (52.9)	1 (25)	0.04
Depression, *n* (%)	46 (28.2)	35 (24.6)	9 (52.9)	2 (50)	0.031
Fatigue, *n* (%)	119 (73)	98 (69)	17 (100)	4 (100)	0.012
Genitourinary, *n* (%)	59 (36.2)	42 (29.6)	13 (76.5)	4 (100)	<0.001
Gastrointestinal, *n* (%)	31 (19)	26 (18.3)	4 (23.5)	1 (25)	NS

Abbreviations: MS—multiple sclerosis; RRMS—relapsing–remitting multiple sclerosis; SPMS—secondary progressive multiple sclerosis; PPMS—primary progressive multiple sclerosis; NS—non-significant.

**Table 5:** Symptoms encountered at the time of the survey in the entire population and according to MS phenotype.

Symptoms	Total Population (*n* = 163)	RRMS (*n* = 142)	SPMS (*n* = 17)	PPMS (*n* = 4)	*p*
None, *n* (%)	72 (44.2)	72 (50.7)	0 (0)	0 (0)	<0.001
Sensory, *n* (%)	24 (14.7)	20 (14.1)	2 (11.8)	2 (50)	NS
Motor, *n* (%)	44 (27)	23 (16.2)	17 (100)	4 (100)	<0.001
Imbalance or discoordination, *n* (%)	28 (17.2)	18 (12.7)	7 (41.2)	3 (75)	<0.001
Blurry vision or ocular pain, *n* (%)	3 (1.8)	3 (2.1)	0 (0)	0 (0)	NS
Diplopia, *n* (%)	4 (2.5)	3 (2.1)	1 (5.9)	0 (0)	NS
Headache, *n* (%)	7 (4.3)	5 (3.5)	2 (11.8)	0 (0)	NS
Trigeminal neuralgia or other facial pain, *n* (%)	6 (3.7)	3 (2.1)	2 (11.8)	1 (25)	0.01
Facial asymmetry or facial spasm, *n* (%)	3 (1.8)	2 (1.4)	1 (5.9)	0 (0)	NS
Vertigo or dizziness, *n* (%)	17 (10.4)	11 (7.7)	5 (29.4)	1 (25)	0.014
Dysarthria, *n* (%)	6 (3.7)	3 (2.1)	1 (5.9)	0 (0)	0.005
Dysphagia, *n* (%)	5 (3.1)	4 (2.8)	1 (5.9)	0 (0)	NS
Hearing impairment, *n* (%)	3 (1.8)	3 (2.1)	0 (0)	0 (0)	NS
Nystagmus-related complaints/oscillopsia, *n* (%)	7 (4.3)	6 (4.2)	0 (0)	1 (25)	NS
Cognitive complaints, *n* (%)	23 (14.1)	16 (11.3)	6 (35.3)	1 (25)	0.023
Depression, *n* (%)	12 (7.4)	8 (5.6)	3 (17.6)	1 (25)	NS
Fatigue, *n* (%)	48 (29.4)	34 (23.9)	12 (64.7)	1 (75)	<0.001
Genitourinary, *n* (%)	25 (15.3)	16 (11.3)	7 (41.2)	2 (50)	0.001
Gastrointestinal, *n* (%)	5 (3.1)	4 (2.8)	1 (5.9)	0 (0)	NS

Abbreviations: MS—multiple sclerosis; RRMS—relapsing–remitting multiple sclerosis; SPMS—secondary progressive multiple sclerosis; PPMS—primary progressive multiple sclerosis; NS—non-significant.

## Data Availability

The dataset presented in this article is not readily available because is part of an ongoing study. Requests to access the dataset should be directed to the corresponding author.
